# Supplementary material for: Simultaneous ligand binding to intact and partially formed ATP-binding sites in the hexameric termination factor Rho
Source: J Biol Chem. 2025 Oct 9;301(12):110797. doi: 10.1016/j.jbc.2025.110797 (PMC12661441; doi:10.1016/j.jbc.2025.110797)
Supplement: Supplementary File [file mmc1.pdf]

Simultaneous ligand binding to intact and partially formed ATP binding sites in the hexameric termination factor Rho

Tyler D Billings[1], Kristie Baker[1], Philip Lacey[1], Matthew Benedek[1], Rodrigo Muzquiz[1], Vicki H. Wysocki[1,2,], and Mark P. Foster[1,*].*

1. Department of Chemistry and Biochemistry, The Ohio State University, Columbus, Ohio 43210
2. School of Chemistry & Biochemistry, Georgia Institute of Technology, Atlanta, Georgia 30318

KEYWORDS. native mass spectrometry, thermodynamics, cooperativity, oligomerization, helicase, Rho

Corresponding Author

*Mark P. Foster

Email address: foster.281@osu.edu

Mailing address: 734 Riffe Building, 496 West 12th Avenue, Columbus, Ohio 43210

Landline: 614-292-1377

Author Contributions

Tyler D Billings: Writing, Conceptualization, Investigation, Resources, Methodology, Formal Analysis, Visualization.

Kristie Baker: Writing, Investigation.

Philip Lacey: Writing, Investigation, Formal Analysis, Visualization.

Rodrigo Muzquiz: Conceptualization, Investigation, Methodology.

Vicki H. Wysocki: Writing, Resources, Supervision, Funding Acquisition.

Mark P. Foster: Writing, Conceptualization, Formal Analysis, Supervision, Funding Acquisition.

Table S 1. Buffers used to prepare and store Rho and its ligands. FPLC buffers were degassed under vacuum.

Buffer Name	Component Concentration	Recipe
**Storage/SEC**	20 mM Tris at pH 7.6<br>200 mM KCl<br>0.2 mM EDTA<br>0.2 mM DTT<br>5% glycerol	• 2.341 Tris HCl, 0.624 g Tris Base<br>• 14.91 g KCl<br>• 0.058 g EDTA<br>• 0.031 g DTT<br>• 50 g glycerol
**Lysis**	50 mM Tris at pH 7.6<br>250 mM KCl<br>1 mM TCEP<br>10% glycerol	• 6.233 g Tris HCl, 1.266 g Tris Base<br>• 18.64 g KCl<br>• 0.250 g TCEP<br>• 100 g glycerol
**Heparin A**	10 mM Tris at pH 7.6<br>0.1 mM EDTA<br>0.1 mM DTT<br>5% glycerol	• 1.17g Tris HCl, 0.312 g Tris Base<br>• 0.029 g EDTA<br>• 0.015 g DTT<br>• 50 g glycerol
**Heparin B**	10 mM Tris at pH 7.6<br>1 M NaCl<br>0.1 mM EDTA<br>0.1 mM DTT<br>5% glycerol	• 1.17 g Tris HCl, 0.312 g Tris Base<br>• 58.44 g NaCl<br>• 0.029 g EDTA<br>• 0.015 g DTT<br>• 50 g glycerol
**ESI Buffer**	100 mM EDDA at pH 7.1	• 17.617 g EDDA

Table S 2. Parameters to manufacture capillary emitters using a P97 micropipette puller.

**Line**	**Heat**	**Pull**	**Velocity**	**Time**
1	R*+45	5	17	100
2	R+40	5	17	100
3	R+35	5	17	100
4	R+25	5	17	100
5	R+20	5	17	100
6	R+15	5	17	1

*R is the ramp temperature, which can change over time or when a new filament is installed.

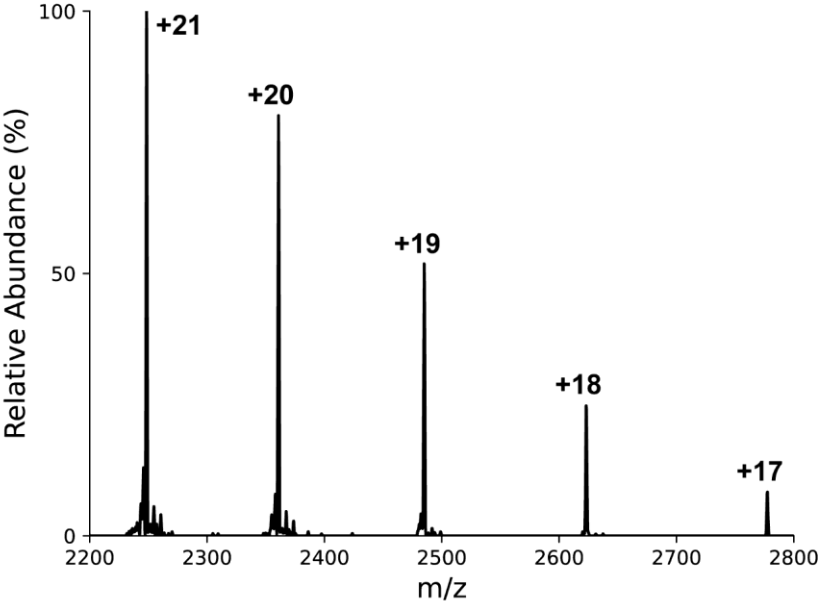

Figure S 1. Intact monomer mass is confirmed from multiple charge states in a mass spectrum generated by activating 2 µM Rho in 300 mM ammonium acetate. Spectrum was recorded using a Thermo Q Exactive UHMR Hybrid Quadrupole-Orbitrap with an in-house SID device in place of the transport multipole.(1, 2) Rho was activated using IST 200 and HCD 200. The high charge is indicative of an unfolded monomer. The peak positions are as follows: 2777.41 (+17), 2623.09 (+18), 2485.03 (+19), 2360.87 (+20), and 2248.49 (+21). The deconvolved mass from these peaks is 47,197.57 ± 0.87 Da. The expected molecular mass post-N-Met processing is 47,198.40 Da. The predicted mass is slightly larger than Rho as found in E. coli strain K-12 (UniProt accession P0AG30, 46,873.02 Da) due to an N-terminal expression tag (Met-Gly-His-EcoRho) incorporated by the Berger group.(3)

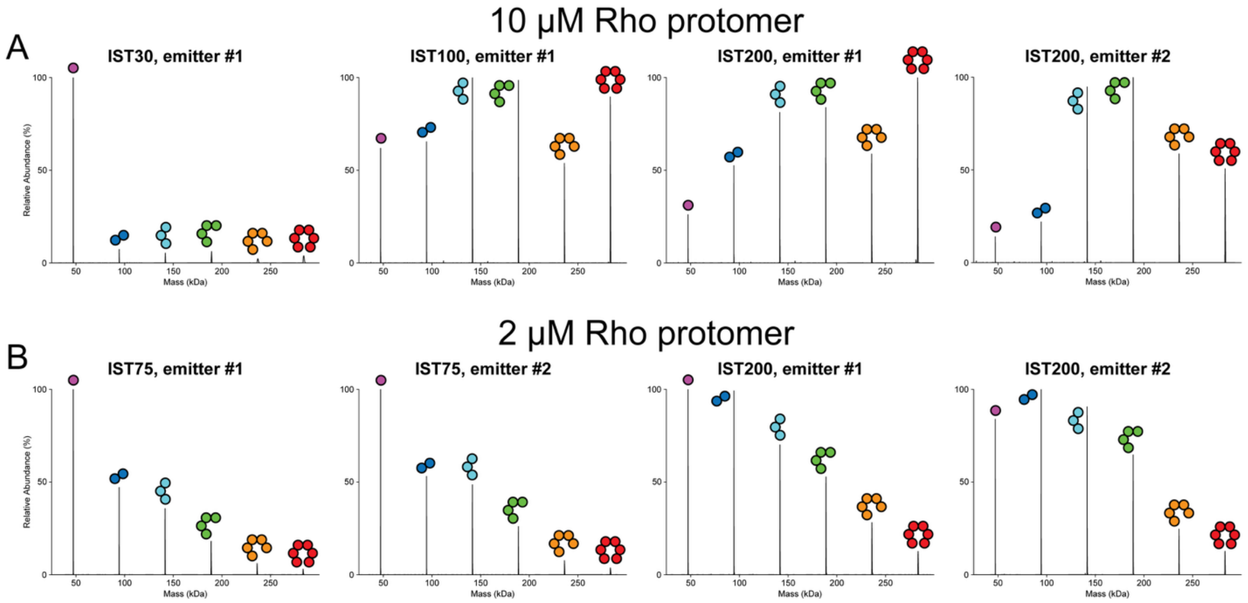

Figure S 2. Signal intensity for different oligomeric states of Rho in native mass spectra was highly variable, independent of concentration. Top row (A) is 10 µM Rho, bottom row (B) is 2 µM Rho. In-source trapping (IST) voltages and emitter ID are labeled for each plot. Populations are derived from UniDec deconvolution(4), with mass range of 25,000 Da to 300,000 Da, automatic m/z peak width determination turned off, Softmax smoothing of 10 and point smooth width of 10. Relative intensities are observed to vary with activation and emitter (capillary tip), making these spectra poor metrics of the population of each oligomeric state.

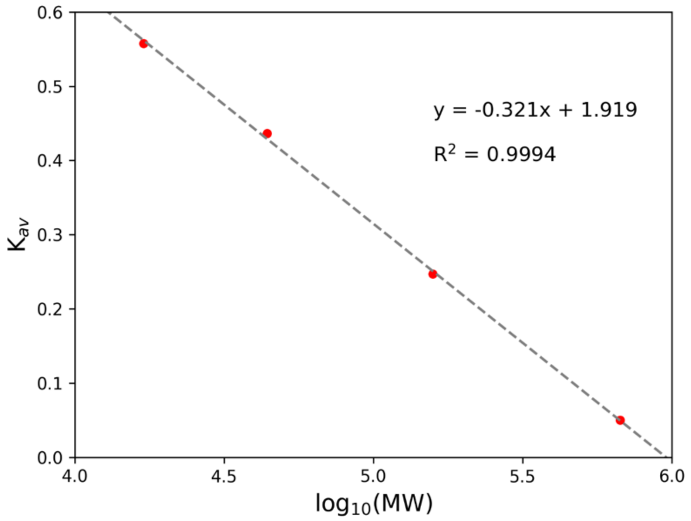

Figure S 3. Calibration curve to predict elution volumes of varying EcoRho oligomeric assemblies. Protein standards are bovine thyroglobulin (670,000 Da), bovine gamma-globulin (158,000 Da), chicken ovalbumin (44,000 Da), and horse myoglobin (17,000 Da). Vitamin B12 (1,350 Da) was excluded from the calibration as its molecular weight lies outside the linear range of the column. K_{av} is determined from:

$$K_{AV} = \frac{(V_e - V_0)}{(V_t - V_0)}$$

where V_e is the specific elution volume of the analyte, V_0 is the void volume of the column, and V_t is the total volume of the column. The regression statistics are reported in the plot.

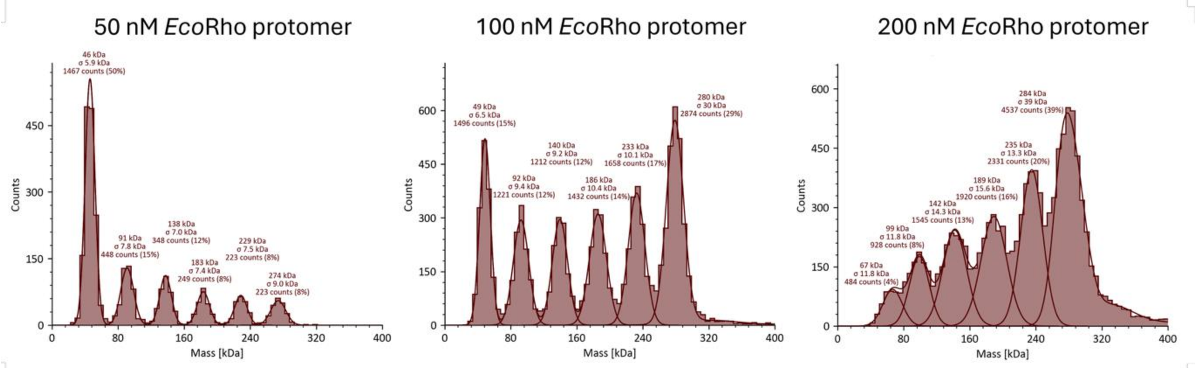

Figure S 4. E. coli Rho oligomerization can be measured by mass photometry. The 100 nM measurement is a replicate collected on a different day from the data presented in Figure 1D. The relative abundance of oligomeric species is suggestive of positively cooperative self-assembly. Protomer concentrations higher than 200 nM were also assayed, but the particle count distributions coalesce to an average molecular weight of 400 ± 100 kDa.

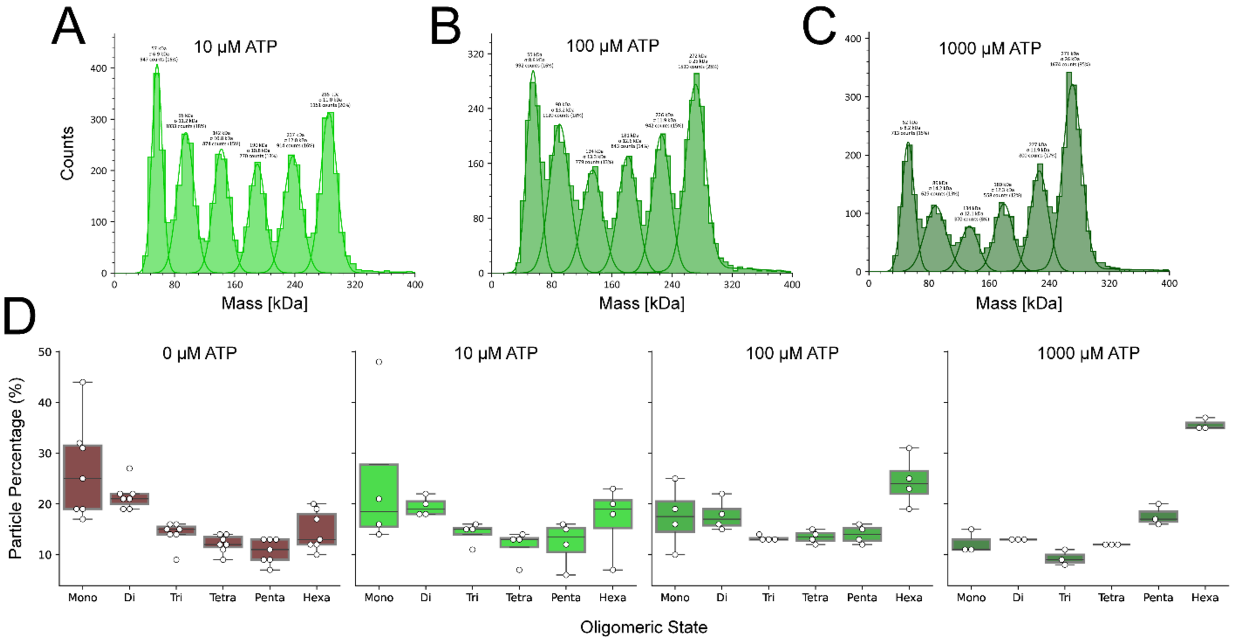

Figure S 5. Mass photometry distributions reporting the percentage of adsorbed particles assigned to monomer, dimer, trimer, tetramer, pentamer, and hexamer species of 100 nM Rho at different ATP concentrations. Representative histograms of particle distributions at 10 (A), 100 (B), and 1000 (C) μM ATP are shown. (D) Box and whisker plots of experimental replicates at the indicated ATP concentrations reveal little difference in oligomeric state distributions from 0-100 μM, but increased counts for the higher oligomeric states at 1 mM ATP.

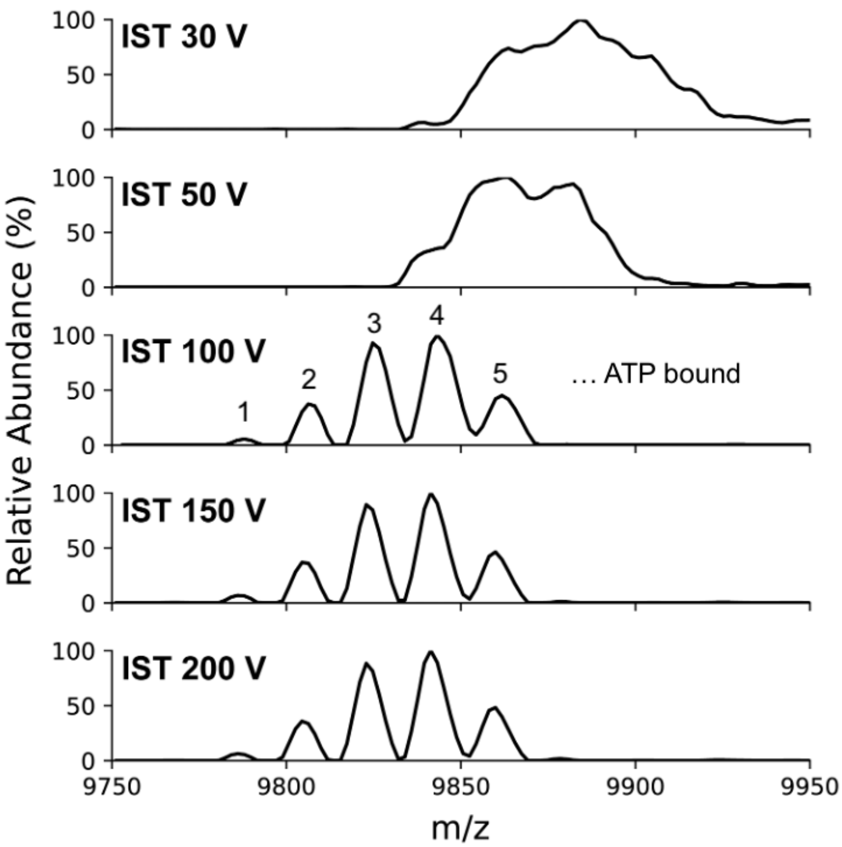

Figure S 6. Moderate in-source trapping does not alter relative intensities of differently ATP-bound Rho. The +29 charge state of the hexamer is used as an example. The peaks in the 100 V panel were assigned to the indicated ATP-bound states shown in Figure 2. 75 V was selected for titration experiments as sub-50 V resulted in poor resolution.

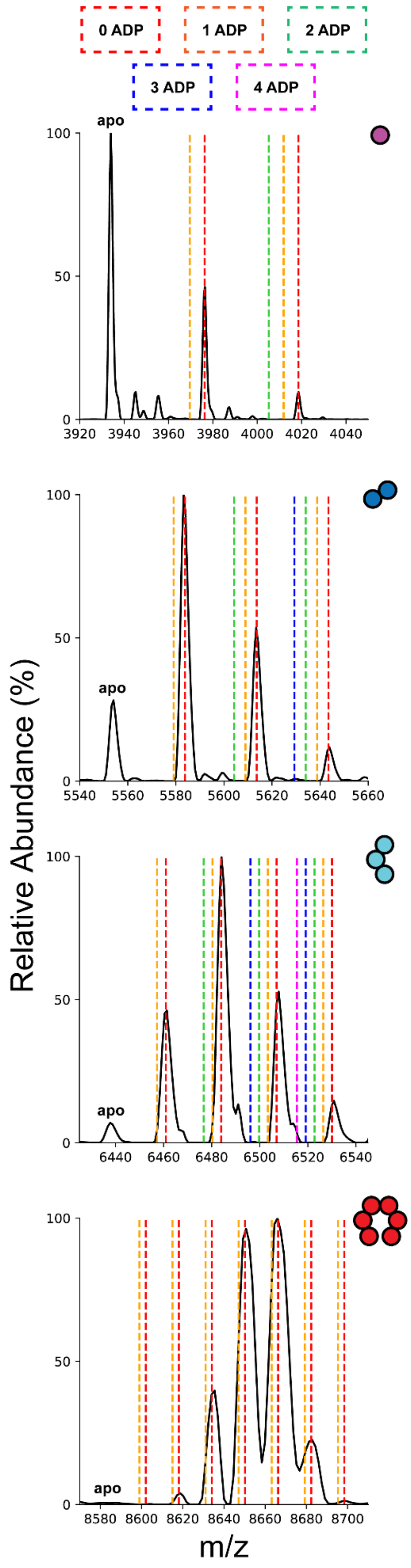

Figure S 7. Predicted combinations of ATP- and ADP-bound mass shifts are labeled on mass spectra of monomer (purple subunit), dimer (blue subunits), trimer (cyan subunits), and hexamer (red subunits) mixed with 100 µM ATP. Dashed lines are color-coded according to the number of ADP molecules for a given combination. Spectra of lower order oligomeric species contain no evidence of ADP binding in place of ATP. Lower resolution between liganded populations makes such interpretation difficult for tetramer through hexamer species, as the peak widths are comparable to the predicted mass difference when substituting ADP for ATP.

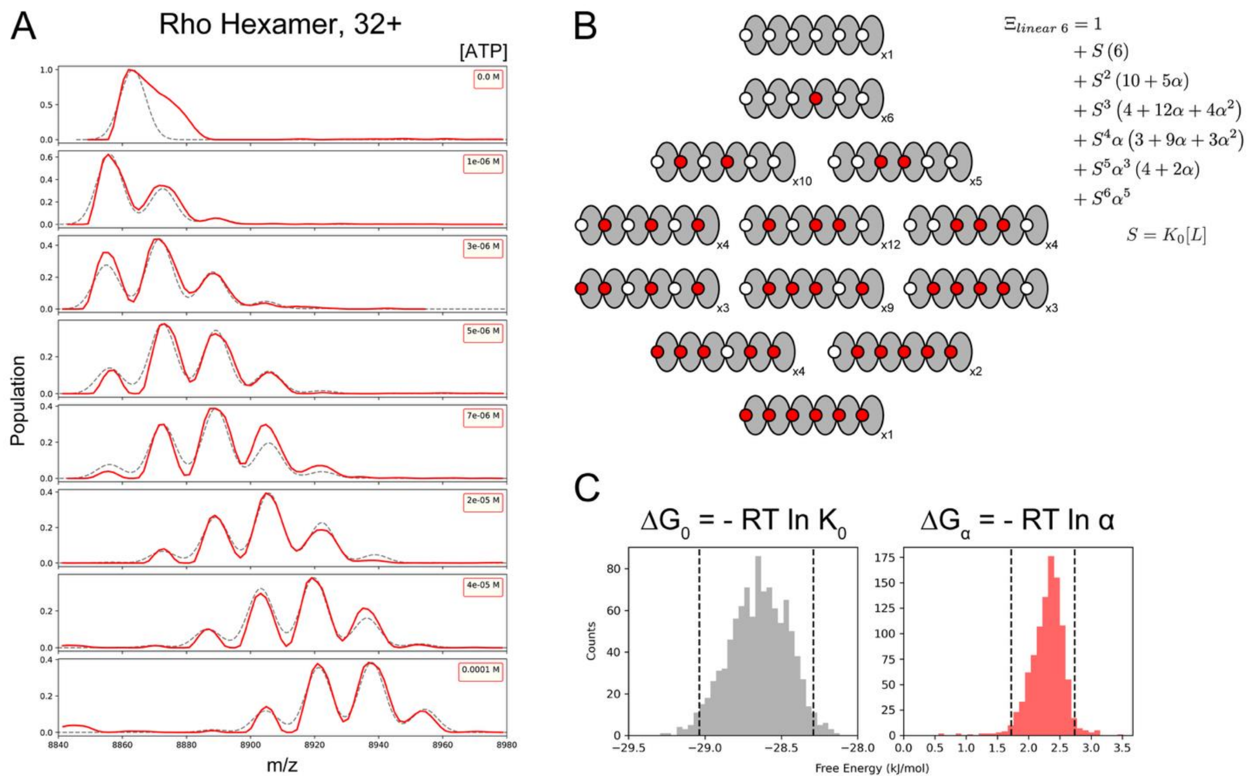

Figure S 8. A negatively cooperative nearest-neighbor model comes close to reproducing experimentally measured Rho-ATP hexamer populations. (A) Region of the nMS spectrum of 10 μM Rho corresponding to the hexameric state, in the absence (top) and presence of increasing ATP concentration, from 1 to 100 μM. Experimental data in red. Dashed lines are simulated spectra generated from optimized parameters from fitting liganded populations to a 6-site linear nearest-neighbor lattice model using itcsimlib.(5, 6) Because the absolute concentration of the hexameric state of Rho could not be determined from the MS data (Figure S2), the lattice concentration was estimated by optimizing goodness-of-fit through a grid search, yielding a minimum at 430 nM hexamer (2.6 μM, or approximately one-fourth of the total protein). (B) Left and right, schematic and polynomial enumerating possible states for binding of a ligand to a linear six-site lattice; numbers indicate degeneracy of states. $S = K_0[L]$, where [L] is the free ligand concentration and K_0 is the equilibrium association constant for binding to an isolated site. The parameter $\alpha = \exp(-\Delta G_\alpha/RT)$ quantifies the thermodynamic interaction between adjacent occupied sites and is 1 for no interaction, >1 for positive cooperativity, and <1 for negative cooperativity. (C) Results from 1000 bootstrap calculations to estimate uncertainty in the fitted parameters. Best fit values correspond to an intrinsic dissociation constant $1/K_0$ = 10 μM (95% CI [8, 11] μM) and negative cooperativity term α = 0.4 (95% CI [0.3, 0.5]).

References

1. Stiving, A. Q., VanAernum, Z. L., Busch, F., Harvey, S. R., Sarni, S. H., and Wysocki, V. H. (2019) Surface-Induced Dissociation: An Effective Method for Characterization of Protein Quaternary Structure. *Anal. Chem.* **91**, 190–209
2. Snyder, D. T., Panczyk, E. M., Somogyi, A., Kaplan, D. A., and Wysocki, V. (2020) Simple and Minimally Invasive SID Devices for Native Mass Spectrometry. *Anal. Chem.* **92**, 11195–11203
3. Lawson, M. R., Ma, W., Bellecourt, M. J., Artsimovitch, I., Martin, A., Landick, R., Schulten, K., and Berger, J. M. (2018) Mechanism for the Regulated Control of Bacterial Transcription Termination by a Universal Adaptor Protein. *Molecular Cell*. **71**, 911-922.e4
4. Marty, M. T., Baldwin, A. J., Marklund, E. G., Hochberg, G. K. A., Benesch, J. L. P., and Robinson, C. V. (2015) Bayesian Deconvolution of Mass and Ion Mobility Spectra: From Binary Interactions to Polydisperse Ensembles. *Anal. Chem.* **87**, 4370–4376
5. Ihms, E. C., Kleckner, I. R., Gollnick, P., and Foster, M. P. (2017) Mechanistic Models Fit to Variable Temperature Calorimetric Data Provide Insights into Cooperativity. *Biophysical Journal*. **112**, 1328–1338
6. Li, W., Yang, H., Stachowski, K., Norris, A. S., Lichtenthal, K., Kelly, S., Gollnick, P., Wysocki, V. H., and Foster, M. P. (2025) Structural basis of nearest-neighbor cooperativity in the ring-shaped gene regulatory protein TRAP from protein engineering and cryo-EM. *Proceedings of the National Academy of Sciences*. **122**, e2409030121
